# Sustainability in interventional radiology: are we doing enough to save the environment?

**DOI:** 10.1186/s42155-022-00336-9

**Published:** 2022-11-28

**Authors:** Pey Ling Shum, Hong Kuan Kok, Julian Maingard, Kevin Zhou, Vivienne Van Damme, Christen D. Barras, Lee-Anne Slater, Winston Chong, Ronil V. Chandra, Ashu Jhamb, Mark Brooks, Hamed Asadi

**Affiliations:** 1 Monash Health, Clayton, VIC Australia; 2 Interventional Radiology Service, Department of Radiology, Northern Health, Epping, VIC Australia; 3 School of Medicine, Faculty of Health, Deakin University, Geelong, VIC Australia; 4 Faculty of Medicine, Nursing and Health Sciences, Monash University, Clayton, VIC Australia; 5 Interventional Neuroradiology Service, Department of Radiology, Austin Health, Heidelberg, VIC Australia; 6 Interventional Radiology Service, Department of Radiology, St. Vincent’s Hospital, Fitzroy, VIC Australia; 7 Epworth Hospital, Richmond, Melbourne, Australia; 8 Interventional Neuroradiology Unit, Monash Imaging, Monash Health, Clayton, VIC Australia; 9 South Australian Health and Medical Research Institute, Adelaide, South Australia Australia; 10 Department of Radiology, Royal Adelaide Hospital, Adelaide, South Australia Australia; 11 Florey Institute of Neuroscience and Mental Health - Austin Campus, Stroke Division, Heidelberg, VIC Australia

**Keywords:** Interventional Radiology, Environmental sustainability, Waste, Climate change, Greening, Carbon footprint, Recycling, Global warming

## Abstract

**Background:**

Healthcare waste contributes substantially to the world’s carbon footprint. Our aims are to review the current knowledge of Interventional Radiology (IR) waste generation and ways of reducing waste in practice, to quantify the environmental and financial impact of waste generated and address green initiatives to improve IR waste management.

**Methods:**

A systematic literature search was conducted in July 2022 using the Medline and Embase literature databases. The scope of the search included the field of IR as well as operating theatre literature, where relevant to IR practice.

**Results:**

One-hundred articles were reviewed and 68 studies met the inclusion criteria. Greening initiatives include reducing, reusing and recycling waste, as well as strict waste segregation. Interventional radiologists can engage with suppliers to reformulate procedure packs to minimize unnecessary items and packaging. Opened but unused equipment can be prevented if there is better communication within the team and increased staff awareness of wasted equipment cost. Incentives to use soon-to-expire equipment can be offered. Power consumption can be reduced by powering down operating room lights and workstations when not in use, changing to Light Emitting Diode (LED) and motion sensor lightings. Surgical hand wash can be replaced with alcohol-based hand rubs to reduce water usage. Common barriers to improving waste management include the lack of leadership, misconceptions regarding infectious risk, lack of data, concerns about increased workload, negative staff attitudes and resistance to change. Education remains a top priority to engage all staff in sustainable healthcare practices.

**Conclusion:**

Interventional radiologists have a crucial role to play in improving healthcare sustainability. By implementing small, iterative changes to our practice, financial savings, greater efficiency and improved environmental sustainability can be achieved.

## Introduction

Global climate change has been labelled as the defining issue of our time by the United Nations (United Nations, 2022). Healthcare contributes enormously to the world’s carbon footprint because it is highly energy intensive, consumes vast resources and produces large amounts of waste (Wyssusek et al. 2019). Hospitals alone produce more than 7,000 tonnes of solid waste a day (Weiss et al. 2016) with operating theatres (OT) contributing up to 20% of total hospital waste (Southorn et al. 2013). In 2009, the National Health Service (NHS) contributed 3.2% of the United Kingdom’s total carbon footprint (Southorn et al. 2013).

Interventional Radiology (IR) is an essential component of modern healthcare. However, as the volume and complexity of IR procedures increase, the waste burden that is generated also increases exponentially. An audit of greenhouse gas generated by a hospital-based IR department in New York found that ~ 23,500 kg CO_2_e (equivalent to burning 9,900 L of gasoline) was emitted over five consecutive weekdays, with an average of 243 kg CO_2_e generated per IR procedure (Chua et al. 2021). Neurointerventional procedures produced an average of 8 kg of waste per case (Shum et al. 2020). Hence, it is important for IR practitioners to reflect on their practice, change and refine practice and engage in green policy to combat the issue of waste and prioritise environmental sustainability. Despite the global focus of environmental sustainability, this has received little attention in the IR literature.

This review aims to (1) identify the nature of waste generated and ways of reducing waste in IR practice; (2) quantify the environmental and financial implications of generated waste and (3) understand potential barriers to implementation of green initiatives.

## Materials and methods

A systematic literature search was conducted in July 2022 using the Medline and Embase databases. Articles focused on waste generation and management in IR practice were targeted, keywords used include “radiology”, “interventional radiology”, “endovascular”, “recycling”, “waste”, “environment friendly”, “sustainability”, “greening”, “cost”, “climate change”, and “global warming”. However, only 22 articles were found related to Radiology, including nine IR articles and two Interventional Neuroradiology (INR) articles. As a result, “operating theatre” and “operating room” were also included to identify relevant articles that could be relevant to IR practice. Medical Subject Headings (MeSH) were applied when searching the databases to include articles with matching content. Articles were limited to full-text articles published in the English language over the past two decades (2000 to 2022 inclusive). Inclusion criteria encompassed articles that were relevant and applicable to IR including conference abstracts. Articles published in non-peer reviewed sources, non-English publications and articles without full-text access were excluded.

## Results

### Study selection

**Fig. 1:**
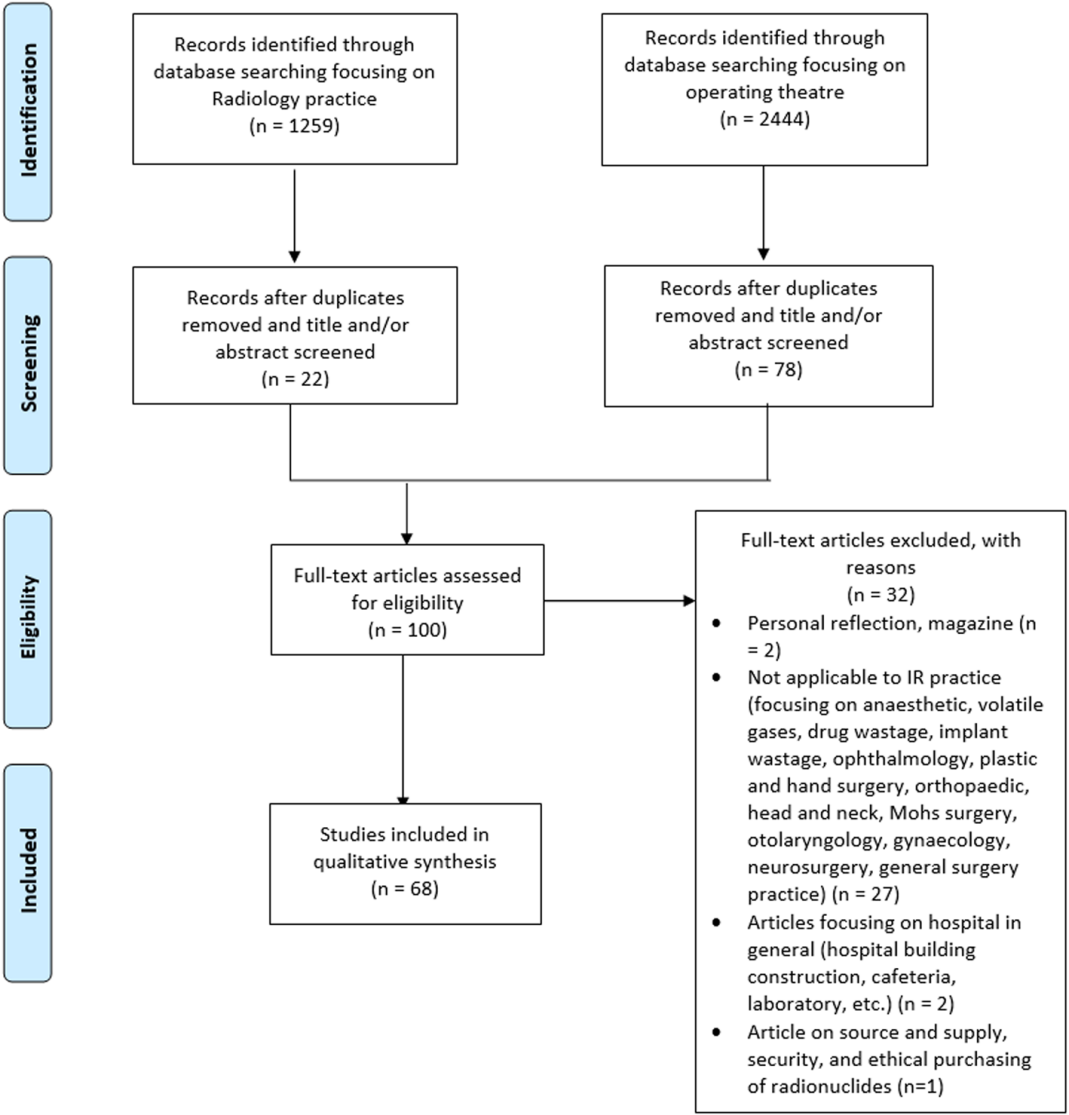
PRISMA flow diagram

One-hundred relevant articles were reviewed. Sixty-eight articles fulfilled the inclusion criteria. We excluded 32 articles due to lack of relevance to IR practice, such as recycling steel from laryngoscope blades, reprocessing single-use device in surgery and recycling in the cafeteria. The selection process and criteria are summarised in a PRISMA (Preferred Reporting Items for Systematic Reviews and Meta-Analyses) flow chart (Fig. 1).

### Study characteristics

The included articles comprised 37 original articles, 13 review articles, 10 editorials, 4 conference articles, 2 commentaries, 1 standard of practice article and 1 presidential address article. All original articles were prospective studies. Thirty-six articles originated from United States, seven from Australia, and the remainder from the United Kingdom, Ireland, Canada, France, Netherlands, India, South Korea, Spain, Switzerland, Turkey and Singapore. Nine articles were related to IR practice, two were related to INR, another ten were related to Radiology and the remainder were related to issues surrounding OT and staff.

The paucity of publications related to IR practice presents a potential publication bias. Most of the original articles consisted of small observational studies or audits and therefore, the reliability of these studies may vary. The heterogeneity of study designs and local waste management protocols also restricts effective comparison of different outcomes. Quality assessment for editorials and standard of practice articles were not performed due to lack of standardised tools.

## Discussion

### Waste composition, segregation and management

Hospital waste can be categorised into general, infectious, pathological (human tissues, organs or fluid), sharps, pharmaceutical, cytotoxic and radioactive waste (Melamed 2003; Wyssusek et al. 2016). General waste is non-hazardous, does not require any treatment and can proceed directly to landfill. By contrast, clinical waste requires treatment prior to safe disposal, which includes incineration, autoclave, microwave, or chemical treatment with or without shredding (Wyssusek et al. 2019). Medical waste is often incinerated (Babu et al. 2019; Yates et al. 2021). Incineration has the benefit of treating all types of waste except radioactive waste, reducing the volume and weight of waste and has the ability of treating large quantities of waste (Kagoma et al. 2012). However, incineration releases toxic fumes (hydrochloric acid, dioxins, and furans) and heavy metals (mercury and lead) into the environment (McLeod 2021; Melamed 2003; Wyssusek et al. 2019). Incineration of 1 kg of clinical waste produces ~ 3 kg of CO_2_ (Southorn et al. 2013). Hence, non-incineration methods should be promoted for medical waste; otherwise, emission from incinerators should be strictly regulated (Melamed 2003).

One study identified that 50–85% of waste in the OT that should have been disposed of as general waste was instead disposed as clinical waste (Kagoma et al. 2012). Waste that was recyclable, such as papers, plastics, cardboards and various wrapping materials was inappropriately disposed of as general waste (Shinn et al. 2017). This suggests a failure in the waste segregation process within clinical environments (Pegg et al. 2022; Wyssusek et al. 2016). Waste misclassification results in suboptimal recycling and an increased cost of up to 20 times to treat and dispose of waste appropriately (Wyssusek et al. 2019) (Table 1).

**Table 1:** Disposal cost for each type of waste

Reference	Cost
Brassil and Torreggiani 2019	Cost of general and clinical waste stream disposal in one of the hospitals in Ireland was €130/tonne (USD 0.145/kg) and €813/tonne (USD 0.907/kg) respectively
McGain et al. 2015	General waste stream disposal (including disposal charge, bag cost, compaction/collection/transport and bin hire) costs AUD 0.24/kg (USD 0.17/kg) whereas clinical waste stream disposal costs AUD 0.98/kg (USD 0.68/kg)
Southorn et al. 2013	In the UK, the cost of waste disposal in 2013 was £0.75/kg (USD 0.98/kg) for sharps, £0.45/kg (USD 0.59/kg) for clinical waste and £0.06/kg (USD 0.078/kg) for general waste
Kagoma et al. 2012	Cost of disposing of solid waste (USD 121 per ton) was twice the cost of recycling plastic (USD 68 per ton)

Reasons for inappropriate waste segregation in clinical bins include lack of understanding about the definition of clinical waste, complacency and fear of reproach (Wyssusek et al. 2019). Examples of items that are often incorrectly misclassified as clinical waste include urinary catheters, suction catheters, oxygen masks and tubing, prep sticks and nasogastric tubes (Perrego 2017). These should not be placed in clinical waste bins unless visibly soiled, dripping or caked with blood or bodily fluids (Beloeil and Albaladejo 2021; Wormer et al. 2013).

Segregation of waste should be done by the person who generated the waste, close to the site of generation (Wyssusek et al. 2019). To encourage waste separation and prevent misclassification, bins should be labelled with clear signage and examples of appropriate waste (Kagoma et al. 2012). By positioning the recycling bin to be readily available at the start of a procedure and placing the clinical waste bin further away, waste is less likely to be inappropriately segregated (Brassil and Torreggiani 2019). Brassil et al. found that waste is 60% more likely to be inappropriately segregated when the risk bin was physically closer to the interventionalist than the non-risk bin during procedures (Brassil et al. 2019). Clinical waste can also be halved if waste generated before the patient entered the OT is separated from the OT waste, because all the waste generated prior to the operation will be general waste (Hubbard et al. 2017; Wyssusek et al. 2019).

### Excess packaging and procedure packs

IR procedures generate a large amount of hospital waste because most devices come with extensive packaging and many are single-use items. Clements et al. measured the weight of a range of IR products including catheters and sheaths, wires, needles, devices, coils and packs/ancillary, and found that the proportion of waste (for packaging or shipping purposes) constituted 54.8% of the overall weight of IR products, of which 76% was potentially recyclable (Clements et al. 2020). In addition, Brassil and Torreggiani found that almost all the packaging from common IR devices were recyclable, with zero cost to the hospital (Brassil and Torreggiani 2019). Prior to the discovery, this packaging was misplaced in the general or clinical waste bins which have disposal costs of €130/tonne (USD 128/tonne) and €813/tonne (USD 801/tonne) respectively (Brassil and Torreggiani 2019). The same authors also found that 12% of peripherally inserted central catheter (PICC) set components and 14% of port set components in the procedure packs were never used and disposed of while additional items were opened to make up the need (Brassil and Torreggiani 2019). An average of 41.5 kg of CO_2_ is produced per PICC insertion procedure (Bolger et al. 2016). By optimising packaging recycling, 35% of the material is salvageable, leading to significant reductions in CO_2_ emissions (Bolger et al. 2016).

It is suggested that IRs engage with suppliers to re-design procedure packs according to local preferences to minimize unnecessary items and packaging (Brassil and Torreggiani 2019). Procedure packs should be reformulated so that items that are not used regularly are removed and packaged individually for occasional use (Van Norman and Jackson 2020). Moreover, packaging can be repurposed for other usage to minimise waste. For instance, Egan and Cheng described a novel way of repurposing Bair Hugger packaging as a keyboard cover to improve OT hygiene and reduce contamination (Egan and Cheng 2012).

### Inventory

IR suites are equipped with a wide range of inventory, some of which are rarely used but kept available for emergencies or unexpected complications. It is not uncommon for devices to remain unused and discarded after their expiry (Demmert and Hong 2019). This increases the financial burden of the hospital and also creates waste. Ways to limit waste associated with expired inventory include adopting the “first in, first out” method, consignment programs or rotating out the materials to the supplier prior to expiration (Baerlocher et al. 2017).

In a large tertiary US-based IR department, incentives (USD 5 coffee gift cards) were given to staff who used soon-to-expire equipment (Demmert and Hong 2019). Over the 13-month investigation period, out of USD 422,732.65 worth of soon-to-expire equipment, USD 135,859.65 worth of equipment was successfully used prior to expiry (Demmert and Hong 2019). Through this incentive program, the department was able to reduce the amount of wasted, expired supplies by almost 50%, with a reduction in the cost of wasted supplies by 31.3% (Demmert and Hong 2019).

Furthermore, we should adopt the practice of environmentally preferable purchasing which prioritises the product’s environmental impact and long-term cost – from production to disposal (Yates et al. 2021). A study found that 72% of suppliers to a university hospital operating room (152 out of 211 companies) do not advertise or promote any sustainability practices in their products, suggesting that hospital procurement departments make purchasing decisions based solely on price or quality (Schieble 2008).

### Opened but unused items

One article investigated the cost of one-time use items opened but not used during neurointerventional procedures (Rigante et al. 2017). Calculating the total costs of unused disposable supply, an average of €676.49 (USD 666.77) per case was wasted for endovascular procedures while €18.44 per case was wasted for diagnostic angiography (Rigante et al. 2017). Aneurysm coiling accounted for the highest waste of €1061.55 (USD 1046.30) (Rigante et al. 2017). Strategies suggested to reduce this include providing education to staff regarding interventional waste, using operator preference cards to provide only necessary supplies, making price transparent to operators, discussing amount of waste during sign out of every procedure, monitoring department inventory and promoting price competition among suppliers by putting the supplier contract out to tender (Rigante et al. 2017; Zygourakis et al. 2017). Other suggestions include opening equipment “just-in-time” for non-emergent cases and having better communication between operators and support staff to prevent equipment expiration (Stall et al. 2013).

Consistent operator-scrub team combinations will optimise team dynamics and improve scrub nurses’ familiarity with operator preferences and practices, leading to more effective communication and correct anticipation of the operator’s needs, thereby preventing the opening of unnecessary items and reducing waste generation (Deshpande et al. 2021). Miscommunication, sterile surgical kits, and overpreparation for potential emergencies were cited as the most frequent causes of OT waste and it is also estimated 26% of single-use, sterile supplies opened during surgery were unused at the end of the case (Meyer et al. 2022).

In addition, unused equipment may be donated to developing countries, medical or veterinary schools, as well as schools for art projects (Wyssusek et al. 2019). Organisations such as REMEDY (Recovered Medical Equipment for the Developing World) at Yale University, InterVol, MedWish and MedShare collect unused medical supplies and ship them to developing countries as humanitarian aid (Chua et al. 2021; Guetter et al. 2018; Weiss et al. 2016).

### Cost

It is important to educate IRs regarding costs incurred in the angiography suite and how they can make a difference by using a less expensive alternative or not using one at all. A cross-sectional online survey showed that IRs and vascular surgeons have limited knowledge regarding the cost of common devices (Wang et al. 2016). Among 1,090 participants who completed the survey, 19.8%, 22.8% and 31.9% were correct in estimating the price of devices, Medicare reimbursement and work relative value unit for procedures (Wang et al. 2016). While almost all respondents indicated they would opt to use cheaper devices, only 24.1% of clinicians had adequate access to hospital pricing information (Wang et al. 2016). Confidentiality clauses that prohibit the disclosure of negotiated prices, monopoly of devices by one supplier due to distinct device characteristics, lack of competitive pricing information and cost transparency are factors which impede clinicians from putting cost into consideration when choosing devices (Wang et al. 2016). Another survey among OT personnel including nurses, surgical technicians, nurse anaesthetists, anaesthesiologists, surgeons and residents found that there is a knowledge deficit around item costs among all OT personnel with only 16.4% of estimates accurate to within 50% of actual price (Heiman et al. 2022).

### Reduce, reuse, recycle

#### Paper

Reduce, reuse and recycle are the key strategies to reduce paper waste. Chawla et al. suggested the use of default double-sided printing for all printers, the use of recycled papers and envelopes, reduced printing of request forms, provision of scrap paper for internal notes and use of digital notepads (Chawla et al. 2017). An audit of waste generated during neurointerventional procedures found that a significant amount of paper, cardboard packaging and user manuals were produced (Shum et al. 2020). These were never read and go straight into the general or recyclable bin (Shum et al. 2020). By digitising these manuals via online internet links or Quick Response codes, the amount of paper waste can be reduced (Shum et al. 2020). On top of that, electronic referrals and medical records can reduce paper volumes (Romero et al. 2012).

#### Co-mingled glass and plastics

Plastics account for a large proportion of recyclable waste; at least 20% of all medical waste is plastic (McCain et al. 2008). Up to 84% of plastics in the OT are potentially recyclable (Wyssusek et al. 2019). Examples of these include polyethylene (Resin Identification code 1 i.e. half-paper/half-plastic instrument wraps, saline and water ampoules, intravenous fluid bags and warming blanket wraps), polyvinylchloride (Resin Identification code 3 i.e. oxygen masks, oxygen tubing, IV fluid bags and giving sets, suction tubing), and polypropylene (Resin Identification code 5 i.e. paper looking surgical instrument wraps, disposable forced air-warming blankets) (McCain et al. 2008; Xiao et al. 2021). Plastic pollution is further aggravated by the high demand for plastic personal protective equipment due to the COVID-19 pandemic.

Hospitals can engage with recycling contractors so that local plastics can convert into furniture, agricultural piping or artwork (McGain et al. 2015). Glass and plastic containers from drugs or contrast can be triple-rinsed and recycled (Schwartz 2010). Soft plastics, include those that are easily scrunched or broken when crushed by hand, such as cling wrap, pallet wrap, plastic bags, bubble wrap, and plastic packaging used for medical equipment, can be recycled (Shum et al. 2020).

#### Blue wrap

Blue wrap is made of polypropylene and is ubiquitous in hospitals to protect patient gowns and toiletries, medical devices and surgical instruments from contamination. Blue wrap contributed to 19% of OT waste and 5% of hospital waste (Babu et al. 2019). Although blue wrap is not reusable or biodegradable, it can be recycled or melted to pellet form and made into other polypropylene items (Babu et al. 2019). Babu et al. launched an 8-week pilot project to recycle blue wrap and was able to collect 1,247 pounds of blue wrap (Babu et al. 2019). By diverting this from landfill, 31.2 cubic feet of landfill space was saved (Babu et al. 2019). Through this project, they extrapolate to yield USD 5,000 in revenue annually and save USD 174,240 from cost avoidance related to reduced disposal and transport costs (Babu et al. 2019). Replacing blue sterile wrap with hard metal cases is another environmentally friendly and cost saving solution (Pradere et al. 2022; Wyssusek et al. 2019). Many volunteers also spearheaded projects to repurpose blue wraps into ponchos, duffel bags, bedrolls, tarps, shopping bags, wallets, neckties, or sleeping bags for charity, for the homeless or distribute them for free (Wu and Cerceo 2021).

### Power consumption

The energy efficiency of Radiology and IR equipment, such as CT, MRI, fluoroscopy and PACS monitors can be improved (Flowers 2020). Heye et al. discovered that the electricity usage of three CT and four MRI scanners over 1 year was adequate to power a town of 852 people (Heye et al. 2020). The energy used for each individual MRI study is similar to the energy consumption for cooling a three-bedroom house with central air conditioning for 1 day or the desalination of 7,000 gallons of fresh water (Buckley and MacMahon 2021). Provided that patient outcomes are not affected, other imaging modalities which utilise less energy such as ultrasound can be chosen to follow up certain diseases rather than MRI (Buckley and MacMahon 2021). Further research is required to promote computationally efficient algorithms and energy efficient hardware (Buckley and MacMahon 2021). In addition, the storage and transmission of large volume of medical imaging data requires energy, especially with increasing complexity of imaging studies being performed (Buckley and MacMahon 2021). Carbon emission from data centres across the world may be comparable to the global aviation industry (Buckley and MacMahon 2021). Clear guidelines should be developed to minimise excess or redundant data that is of no perceived future benefit (Buckley and MacMahon 2021).

An energy audit in an Irish teaching Radiology department found that 29 of 43 desktop computers and 25 of 27 PACS reporting stations were left unnecessarily powered afterhours (McCarthy et al. 2014). Hainc et al. evaluated the power consumption of 36 reporting workstations in a Radiology department. The on-mode consumption per year was 40,763 kWh/a, the stand-by-mode consumption was 10,010 kWh/a and the off-mode consumption was 2,397 kWh/a (Hainc et al. 2020). Power consumption can be reduced by using the auto-shutdown function in computers and PACS monitors or using energy saving computers (Chawla et al. 2017). 45% of power consumption can be reduced if the stand-by mode of 4 h wait time was skipped, and the wait-time for shutdown was set to one hour (Hainc et al. 2020). By shutting down workstations and monitors after an 8-hour workday, the Radiology department would save 83,866.6 kWh of electric consumption and USD 9,225.33 annually (Prasanna et al. 2011). This is equivalent to removing 11.6 cars off the road, saving 14.9 barrels of oil or 39 tonnes of coal, thereby reducing associated greenhouse gases and carbon emissions (Prasanna et al. 2011). During working hours, monitors should go into sleep mode if not in use for more than 20 min (Schwartz 2010).

A systemic review found that electricity use constitutes the major carbon footprint within the OT (Hainc et al. 2020; Rizan et al. 2020). Ways to improve energy efficiency of OTs include installing occupancy sensors, low energy lighting and energy efficient air conditioning systems (Hainc et al. 2020). The largest source of CO_2_ emission comes from electricity and gas used to power the climate control system in the IR suite (Chua et al. 2021). Of note, more than half of the heating, ventilation and air conditioning (HVAC) energy use was generated during off hours while the IR suite was rarely in use (Chua et al. 2021). By turning off the HVAC systems in unused OTs during after hours, MacNeill et al. found that energy consumption was reduced by half (McLeod 2021). Another idea is to implement HVAC setback in the OTs, an energy-saving strategy that reduces the frequency of fresh air exchanges to the OTs and allows temperature and humidity settings to fluctuate when not in use or at night (Chua et al. 2021; Saver 2011; Yates et al. 2021). The Cleveland Clinic saved USD 2 million a year by reducing air exchanges from 20 times an hour to 6 times an hour when OTs are not in use (McLeod 2021).

In addition, incandescent bulbs should be replaced with light emitting diode (LED) lamps as they have longer life, are more energy efficient and do not generate unwanted heat or radiation (Chawla et al. 2017; Yates et al. 2021). LED lamps can extend the lifetime of an OT light from 1 to 6 years of continuous use, resulting in long term cost savings (Yates et al. 2021). An audit of 18 OTs in 11 hospitals in Turkey found that 88.3% of the OTs did not use sensor controls on lights and 66.7% did not use LED lights (Dönmez et al. 2019). A teaching hospital in Oregon saved approximately 340,000 kilowatt-hours (kWh) of energy annually, which is equivalent to saving USD 40,000 per year, just by refitting the OTs with LED lights and low-mercury lamps (Saver 2009). By powering down all anaesthesia and OT lights and equipment not in use, USD 33,000 was saved and annual CO_2_ emissions were reduced by 234.3 metric tonnes (Wormer et al. 2013). This can also be achieved via switching to motion activated lighting (Chawla et al. 2017).

### Electronic devices and machines

Radiology is a technology-centric specialty where the use of machines and electronic equipment is essential. As a result, the disposal of this large number of electronic devices becomes an environmental issue when they reach the end of their lifespan. Instead of disposing of electronic devices and machines such as computers, scanners, projectors, telephones, printers, toner cartridges, cables, plugs and batteries, many manufacturers offer recycling programs where these devices can be returned and recycled to make new products (Chawla et al. 2017; Schwartz 2010). We should choose to purchase products from manufacturers who have strict green policies and are committed to recycling and reusing their products, if not disposing of their expired equipment in an environmentally friendly way (Beloeil and Albaladejo 2021; Chawla et al. 2017; Laustsen 2010; Saver 2011).

### Teleradiology and remote working

The COVID-19 pandemic has resulted in the change of working practices across the world due to the need for social distancing. Radiology has an edge over other medical specialties because it offers remote working and tele-radiology as an option, therefore reducing long-distance travel and mitigating the carbon emission to the environment. Radiology trainees travel long distances to their placement sites. Peters et al. found that each trainee in the United Kingdom travelled 6703 miles per year in a car for work or training purposes (Peters et al. 2021). With the introduction of flexible on-call sites whereby Radiology trainees can base themselves at any site to deliver overnight on-call radiology services, the average distance travelled in a car per trainee per year reduced to 6600 miles (103 miles less per trainee) (Peters et al. 2021). Accounting for all 64 Radiology registrars in Plymouth, United Kingdom, this new setup resulted in reduction of 4.3 tonnes CO_2_ emissions, equivalent to 2.6 return flights from London to New York (Peters et al. 2021). Reducing commuting distances has the added benefits of reducing travel expenses, improving safety and freeing up time which could be spent on clinical duties (Peters et al. 2021). In addition, teleradiology can be taken advantage of to provide remote reporting, remote report checking, remote teaching and teleconferencing with multidisciplinary teams to reduce unnecessary travel, hence, improving the environmental sustainability of Radiology training (Peters et al. 2021).

### Water

Alcohol-based hand rubs should be used in between cases for surgical scrubbing rather than water unless visibly soiled to reduce water and drying towel waste (Wyssusek et al. 2019). This practice is supported by the UK National Institute for Health and Care Excellence (NICE) guidelines on preventing surgical site infections (Wyssusek et al. 2019). Surgical hand wash for one staff member during a standard 3-minute period used up 18.5 L of water compared to an average volume of 15 ml alcohol rub (Jehle et al. 2008). By swapping from scrub soap to alcohol-based waterless scrub solutions, Wormer et al. estimated that 2.7 million litres of water could be saved a year (Wormer et al. 2013). For non-surgical hand wash, using hand dryers or hand sanitisers can reduce paper towel waste (McGain et al. 2015).

Different tap designs also contributed to the volume of water waste. Foot pedal systems had the least water usage per scrub (6.7 L), followed by 45 s timed motion triggered sink (7.5 L) and high flow elbow lever tap sink (11 L) (Weiss et al. 2016). A hospital in the US managed to save USD 13,750 per year in water usage costs by installing water saving retrofits throughout their OT (Weiss et al. 2016).

### Reusable sharp containers, surgical linens, and medical supply

Sharp disposal commonly occurs in single-use protective containers. By changing to reusable sharp containers, Saver reported an annual saving of USD 70,000 (Saver 2011).

The production and delivery of disposable surgical supplies constitutes the second greatest source of greenhouse gas emission in the IR suite (9640 kg CO_2_e) (Chua et al. 2021). Items that can be safely reprocessed such as surgical instruments and gowns can be reused to reduce the need of single use supplies (Chua et al. 2021). Disposable surgical linens amounted to 2% of all hospital waste (Stall et al. 2013; Wyssusek et al. 2019). Surgeons can preference reusable surgical gowns, drapes and linens rather than disposables (Guetter et al. 2018; Wyssusek et al. 2019). Disposable clothing has greater environmental impact than reusable clothing (Beloeil and Albaladejo 2021) because reusable gowns utilise 28% less energy, generate 30% less greenhouse gases and consume 50% less water (Beloeil and Albaladejo 2021).

WHO reported no difference in surgical site infections or wound contamination between reusable and disposable equipment (Guetter et al. 2018). By switching to reusable surgical gowns, one hospital reduced 63,000 kg of waste and saved USD 38,000 (Conrardy et al. 2010). Most studies support that the use of reusable generates less waste, has a lower landfill and incineration burden and costs less money in the long run, compared to disposable products (Conrardy et al. 2010; Weiss et al. 2016).

There is increasing need to find reusable medical equipment given the recent shortages caused by the COVID-19 pandemic (Blough and Karsh 2021). Life cycle of medical supply, reusability, recyclability in addition to functionality and safety should be taken into account in product design using next generation materials (Arepally et al. 2022; Blough and Karsh 2021). Comprehensive life cycle assessment of medical devices should be conducted by relevant industry to evaluate the environmental footprint of the products (Baboudjian et al. 2022).

### Barriers

Although most healthcare staff are supportive of waste management and greening strategies, there are a few reported barriers that hamper staff behavioural change and implementation of green initiatives. Common barriers include.

- Lack of leadership

As healthcare staff are compelled to follow their hospital’s policies and guidelines concerning waste management, lack of guidelines and poor implementation of policies make it difficult for staff to embrace green initiatives and culture (Wyssusek et al. 2019). Decisions for changing the culture and engaging in greening policies for hospitals need to come from leaders and senior management (Arepally et al. 2022; Benzil 2019; Kagoma et al. 2012).

- Lack of knowledge and misconceptions regarding infectious risk (Wyssusek et al. 2019)

A survey of 524 hospital surgical staff found that 56.7% participants were uncertain about which OT items are recyclable, the majority attributed lack of knowledge as the greatest barrier to recycling (Azouz et al. 2019). Ongoing staff education and training is essential so that staff know how to segregate waste appropriately, not only to reduce waste, but to prevent contaminated items from being disposed into recycling or general waste bins. This could be done via annual waste training requirements as part of the health and safety training or using posters and signs to increase awareness regarding waste issues (Melamed 2003). A few articles have achieved success in staff education with post education audits showing between 41 and 77% reduction in waste (Perrego 2017; Southorn et al. 2013; Treggiari 2017).

- Lack of data (Wyssusek et al. 2019).
- Lack of awareness, concern, and time to address waste (Meyer et al. 2022)

66.7% of the OTs in 11 hospitals in Izmir Province, Turkey had no environmental team (Dönmez et al. 2019). A survey showed that only 8.4% of respondents received sustainability education as part of their medical student or resident curriculum (Ard et al. 2016).

- Concerns about increased workload, perceived logistical issues and inconvenience (Wyssusek et al. 2019)

There is concern that the need to segregate waste and determine which individual plastic is recyclable will interrupt the workflow of busy OT environments (McCain et al. 2008). If segregation takes place once removed from the OT, this will increase workload and incur additional cost (McCain et al. 2008).

- Staff attitudes and resistance to change

Two surveys among anaesthesiologists demonstrated that although the majority of respondents were interested and willing to commit their time to recycling, recycling only occurred in 11% and 28% of their OTs (Ard et al. 2016; McGain et al. 2012). Another cross-sectional study evaluating knowledge, attitude and practices regarding biomedical waste management among OT personnel in a tertiary hospital found that satisfactory attitude score did not translate into satisfactory knowledge or better practices, hence, highlighting the need for appropriate training (Aanandaswamy et al. 2019).

- Manufacturers and regulatory agencies

An online survey of cataract surgeons and nurses found that 93% of participants believed OT waste is excessive and commonly cited reasons for it were rigid requirements imposed by manufacturers and regulatory agencies on reusing devices, supplies and pharmaceuticals (Chang and Thiel 2020). Most believe that manufacturers are driven to produce more single-use products due to increase profit, liability reduction and lack of carbon footprint considerations (Chang and Thiel 2020).

### Staff education and awareness

It is crucial to inform healthcare providers regarding how their practices affect the environment and the need to take responsibility for minimsing this impact. Radiology as a department can measure their carbon footprints, aiming to achieve a “green” accreditation through appointing sustainability officers to monitor, educate, advocate and implement sustainable initiatives and policies (Arepally et al. 2022). Formation of a hospital green team with participants from different discipline including nursing, anaesthesiology, material managements, environmental services and marketing can drive positive healthcare impact on the environment (Wu and Cerceo 2021). Upchurch suggested scorecards to be used for tracking waste, advocating for incentives for surgeons in governing sterile supplies (Upchurch 2022). Similar to the ALARA (as low as reasonably achievable) principle for radiation exposure, benchmark for carbon footprint should be set to decarbonize radiology (Arepally et al. 2022). A dedicated waste segregation educational programme should be implemented similar to the radiation protection programmes that are already implemented (Pradere et al. 2022) (Table 2).

**Table 2:** Financial and environmental benefits of green initiatives

Reference	Topic	Financial and environmental benefits
Peters et al. 2021	Flexible on-call site for radiology trainees	Reduce commuting distance and resultant reduction in CO_2_ emissions by 4.3 tonnes of CO_2_
McLeod 2021	Recycling medical plastics	Disposing medical plastics as solid waste costs USD 121 per ton compared to USD 68 per ton recycling same material
	Reducing air exchanges when operating rooms are not in use	Saved USD 2 million a year
	Installing HVAC occupancy sensors in 2 operating rooms	Reduced 25,000 kW per hour energy use and saved USD 4,000 dollars per year
Hainc et al. 2020	Changing the wait period of reporting stations stand-by from four hour to one hour, and then turning the reporting stations off completely	Estimated annual saving of 23, 692 kWh, equivalent of saving 45% of initial power consumption
Brassil and Torreggiani 2019	13.5% of general waste consisted of recyclable materials and 16.5% of clinical waste comprised of packaging and paper that could be diverted to recycling stream	391 tonnes of materials could have been recycled, saving hospital waste management costs of up to €33,000 (USD 32,526)
Demmert et al. 2019	Incentives (USD 5 coffee gift cards) were given to staff who used soon-to-expire equipment	USD 135,859.65 worth of equipment was successfully used prior to expiry
Azouz et al. 2019	Implement recycling educational program	Saved 10.3% in sharps waste disposal cost, cost decreased from USD 665.28 to USD 596.66
Babu et al. 2019	Recycling blue wrap over 39 days	Saved 31.2 cubic feet of landfill space
		Extrapolated to yield USD 5,000 in revenue each year and save USD 174,240 from cost avoidance related to decreased disposal and transport costs
		Estimated to save 18,837.5 gallons of oil, equivalent to 158,786 kWH of energy across the whole hospital
Hubbard et al. 2017	Disposing of anaesthesia-related waste in standard waste bins prior to patient entry into operating room	Annual reduction of 13,800 kg of regulated medical waste and a cost savings of USD 2,200
Perrego 2017	Mandatory online learning module for all perioperative staff members	41% reduction in total mass of regulated waste and 77% reduction in total mass of non-regulated items. This translated to an estimated USD 7,200 reduction in regulated waste cost per annum
Wyssusek et al. 2016	Segregating clinical waste from general waste	Approximately 60% reduction of waste disposal costs for operating room waste and saving of USD 5,790 per month
	Further implementation of recycling	Increased overall cost reduction of approximately 80%, which was approximately USD 7,800 in savings per month
Weiss et al. 2016	Installing water saving retrofits throughout OT	Saved USD 13,750 per year in water usage costs
Albert and Rothkopf 2015	Initiating single-stream recycling	Three campuses saved USD 3,487 per month, extrapolating to a saving of USD 41,844 per year
Wormer et al. 2013	Powering down all anaesthesia and OT lights and equipment not in use	Saved USD 33,000 and reduced 234.3 metric tonnes of carbon dioxide emissions per year
	Reusing batteries	Saved USD 9,238 and 500 pounds of alkaline waste was diverted from landfills
	Swapping from scrub soap to alcohol-based waterless scrub solutions	Estimated 2.7 million litres of water saved
Southorn et al. 2013	Recycling and segregating waste generated from joint replacement procedures appropriately	Estimated 75% carbon footprint reduction via reduction of incineration of misclassified clinical waste nationwide in UK
		Estimated £420,000 (USD 474,967) cost saving per year
Prasanna et al. 2011	Shutting down workstations and monitors after an 8-h workday in the radiology department	Saved 83,866.6 kWh of electric consumption and USD 9,225.33 annually. Equivalent of removing 11.6 cars off the road, saving 14.9 barrels of oil or 39 tonnes of coal
Saver 2011	Changing to reusable sharp containers	Annual saving of USD 70,000
Conrardy et al. 2010	Recycling surgical towels, gowns, table covers, stainless steel basins and Mayo stand covers	Reduced waste generation by 45,000 lb and save USD 12,600 from regulated medical waste disposal cost in a hospital that conducted 10,000 surgical procedures a year
	Switching from disposable to reusable surgical gowns	Cut down USD 38,000 and reduced 63,000 kg of waste
Saver 2009	Refitting the OR with LED lights and low-mercury lamps	Saved approximately 340,000 kilowatt-hours (kWh) of energy annually, which is equivalent to saving USD 40,000 per year
	Substituting conventional electricity with clean and sustainable sources of energy via getting Green Tag renewable energy certificate	Reduced carbon emission by 265,000 lb per month
McGain et al. 2008	Recycling plastics from the operating room	Saved AUD 20 (USD 13) per week from recycling 200 kg of plastics

## Conclusion

As demonstrated by the limited number of publications on green initiatives in IR, efforts to tackle this issue in radiology practice need to improve. In the face of climate change, we encourage everyone to discuss environmental awareness in their day-to-day conversation and actively contribute to the global green movement by delivering practical actions to clinical practice (Table 3).

**Table 3:** Summary of green initiatives in IR

WASTE WAR
W aste segregation
A wareness of cost
S taff education
T urn it off. Save energy and water
E lectronic and device recycling
W ait! No opened, unused items
A ssess inventory
R educe, Reuse, Recycle
- Packaging, paper, plastic, glass
- Blue wrap
- Reusable surgical linen
- Reusable sharp containers

## Data Availability

Not applicable.

## Abbreviations

IR: Interventional Radiology
OT: Operating Theatre
INR: Interventional Neuroradiology
PICC: Peripherally inserted central catheter
HVAC: Heating, Ventilation and Air Conditioning
LED: Light Emitting Diode
